# *In Vitro* Treatment of Melanoma Brain Metastasis by Simultaneously Targeting the MAPK and PI3K Signaling Pathways

**DOI:** 10.3390/ijms15058773

**Published:** 2014-05-16

**Authors:** Inderjit Daphu, Sindre Horn, Daniel Stieber, Jobin K. Varughese, Endy Spriet, Hege Avsnes Dale, Kai Ove Skaftnesmo, Rolf Bjerkvig, Frits Thorsen

**Affiliations:** 1 NorLux Neuro-Oncology Laboratory, Department of Biomedicine, University of Bergen, 5009 Bergen, Norway; E-Mails: inderjit.daphu@adm.uib.no (I.D.); sindre.horn@student.uib.no (S.H.); jobinv@gmail.com (J.K.V.); kaioveskaftnesmo@gmail.com (K.O.S.); rolf.bjerkvig@biomed.uib.no (R.B.); 2 NorLux Neuro-Oncology Laboratory, the Luxembourg Public Research Center for Health, 1445 Strassen, Luxembourg; E-Mail: stieberd@mac.com; 3 Molecular Imaging Center, Department of Biomedicine, University of Bergen, 5009 Bergen, Norway; E-Mails: endy.spriet@biomed.uib.no (E.S.); hege.dale@biomed.uib.no (H.A.D.); 4 KG Jebsen Brain Tumour Research Centre, Department of Biomedicine, University of Bergen, 5009 Bergen, Norway

**Keywords:** melanoma brain metastasis, BRAF, PTEN, PI3K (phosphoinositide 3-kinase), MAPK (mitogen-activated protein kinase), mTOR, temsirolimus, vemurafenib

## Abstract

Malignant melanoma is the most lethal form of skin cancer, with a high propensity to metastasize to the brain. More than 60% of melanomas have the BRAF^V600E^ mutation, which activates the mitogen-activated protein kinase (MAPK) pathway [1]. In addition, increased PI3K (phosphoinositide 3-kinase) pathway activity has been demonstrated, through the loss of activity of the tumor suppressor gene, PTEN [2]. Here, we treated two melanoma brain metastasis cell lines, H1_DL2, harboring a BRAF^V600E^ mutation and PTEN loss, and H3, harboring WT (wild-type) BRAF and PTEN loss, with the MAPK (BRAF) inhibitor vemurafenib and the PI3K pathway associated mTOR inhibitor temsirolimus. Combined use of the drugs inhibited tumor cell growth and proliferation *in vitro* in H1_DL2 cells, compared to single drug treatment. Treatment was less effective in the H3 cells. Furthermore, a strong inhibitory effect on the viability of H1_DL2 cells, when grown as 3D multicellular spheroids, was seen. The treatment inhibited the expression of pERK1/2 and reduced the expression of pAKT and p-mTOR in H1_DL2 cells, confirming that the MAPK and PI3K pathways were inhibited after drug treatment. Microarray experiments followed by principal component analysis (PCA) mapping showed distinct gene clustering after treatment, and cell cycle checkpoint regulators were affected. Global gene analysis indicated that functions related to cell survival and invasion were influenced by combined treatment. In conclusion, we demonstrate for the first time that combined therapy with vemurafenib and temsirolimus is effective on melanoma brain metastasis cells *in vitro*. The presented results highlight the potential of combined treatment to overcome treatment resistance that may develop after vemurafenib treatment of melanomas.

## 1. Introduction

Malignant melanomas arise from the transformation of melanocytes, and DNA damage of melanocytes from ultraviolet radiation is regarded as the most important environmental risk factor [3]. The tumors have the highest propensity to metastasize to the brain, seen in around 50% of the melanoma patients with advanced disease [4]. Current treatment strategies of melanoma brain metastasis involve surgery, chemotherapy, radiotherapy and radiosurgery. Treatment effects are, however, minimal, as multiple brain metastases are often found at the time of neurological presentation, which decreases the prognosis considerably [5]. Furthermore, the melanomas are regarded to be highly radioresistant [3]. Further, the blood-brain barrier (BBB) prevents effective drug delivery to the tumors [6], as most of the chemotherapeutics used today are either too large or hydrophilic to cross the BBB [4]. Thus, new therapeutic approaches are warranted, such as using small-sized targeted molecules that may cross the BBB more easily.

The mitogen activated protein kinase (MAPK) and the phosphoinositide 3-kinase (PI3K) signaling pathways are often activated in melanomas by genetic alterations [7]. The MAPK pathway plays a key role in modulating melanoma survival, proliferation, invasion and angiogenesis. In normal cells, this pathway is tightly regulated by extracellular signals from the cell membrane to the nucleus, mediated by a cascade of phosphorylation events. Frequent somatic point mutations have been found in the BRAF (66%) and N-RAS (15%) genes [8–10]. Around 80%–90% of the BRAF mutations display a valine to glutamic acid substitution (BRAF^V600E^) [11], although other activation mutations are also seen, such as BRAF^V600K^ and BRAF^V600R^ [12,13]. Around 69% of the BRAF^V600E^ mutations are reported to be homozygous [14]. The activated RAF kinase phosphorylates and activates the MAPK/extracellular signal-regulated kinases (ERK1/2; MEK1/2), further phosphorylating and activating MEK, which, in turn, phosphorylates and activates ERK1/2. Gene expression, metabolism and cytoskeletal functions are then regulated by ERK1/2 [11,15,16].

The activity of the PI3K pathway is also increased in melanomas, due to the loss of function of the phosphatase and tensin homolog (PTEN), either through mutation, deletion or methylation. PTEN is a critical negative regulator of AKT by preventing its phosphorylation, with subsequent inhibition of the PI3K pathway [17]. Loss of PTEN function has been observed in 10% to 30% of melanomas and is commonly associated with the BRAF^V600E^ mutation [13,18,19]. Increased activity in the PI3K pathway has further been related to an acquired resistance to BRAF and MEK inhibitors after therapy [20]. Thus, a combined inhibition of the MAPK and PI3K signaling pathways in treating melanoma could potentially increase the therapeutic efficacy.

Vemurafenib (PLX4032) is a serine threonine kinase inhibitor specifically targeting mutated BRAF and has recently been approved for treating BRAF^V600E^ mutated melanomas by the US Food and Drug Administration (FDA) [12]. The penetrance of the drug into the human brain has, to our knowledge, not been investigated, but results from a study performed by Mittapalli and colleagues suggested the penetration of vemurafenib into the mouse brain [21]. The drug has shown encouraging results in clinical trials on advanced melanoma [12], although acquired treatment resistance has so far been a major issue [22,23]. Interestingly, there is a few reports in the literature on the treatment of brain metastases using vemurafenib [24–28] and also indicating increased patient survival [24,27] and tumor necrosis [28] after treatment.

Temsirolimus (CCI-779) is an ester derivative of sirolimus (rapamycin), inhibiting mTOR kinase activity. By binding to mTOR, which is downstream of PTEN, signal transduction in the PI3K pathway required for progression through the cell cycle is inhibited [2]. It has previously been reported that therapeutic concentrations of temsirolimus can be achieved in glioblastoma [29]. In a clinical phase II trial using temsirolimus in metastatic melanoma, only a minimal effect was seen, indicating that the drug may not be sufficiently active as a single agent [2]. However, in a murine model, temsirolimus enhanced the anti-tumor activity of cancer vaccines used to treat established renal cell carcinoma and melanoma [30]. This indicates that combined treatment with other drugs could be more effective.

The main purpose of the present work was to study whether combined treatment using vemurafenib and temsirolimus could inhibit the growth of human melanoma brain metastasis cells *in vitro*. To our knowledge, these two drugs have not been combined to treat melanoma brain metastasis cells before. In this study, we used cell lines previously derived from human melanoma brain metastases. Results in our lab suggest that these cells have the ability to specifically home to the mouse brain [31], thus making them a unique tool for studying the therapeutic effects of vemurafenib and temsirolimus on brain metastatic cells *in vitro* and, also, in later *in vivo* studies. Our *in vitro* results show that cell proliferation and migration may be inhibited when the two drugs are used in combination. Moreover, the combined treatment led to reduced pERK1/2, p-mTOR and pAKT activity. Global gene expression analysis indicated that several cellular functions were altered by combined treatment affecting the cell cycle, cell death and survival, cellular movement and DNA-replication, as well as DNA recombination and repair.

## 2. Results and Discussion

### 2.1. BRAF and PTEN Status of the H1_DL2 Melanoma Brain Metastasis Cell Line

Bidirectional DNA sequencing of the H1_DL2 cells showed that the BRAF mutation was heterozygous by a single mutation at exon 15 (nucleotide 1799) of the BRAF gene. This thymidine (T) to adenine (A) transversion mutation results in the substitution of valine with glutamate in codon 600 (V600E) (Figure S1A, red arrow). Furthermore, DNA copy number analysis showed that the H1_DL2 cell line had a homozygous deletion of PTEN on chromosome 10 (Figure S1B,C, red arrows). This indicates that both the MAPK and the PI3K pathways may be activated in these cells.

### 2.2. Treatment with Vemurafenib and Temsirolimus Induces Anti-Proliferative Effects in H1_DL2 and H3 Cell Lines Grown as Monolayers

**Figure 1.**
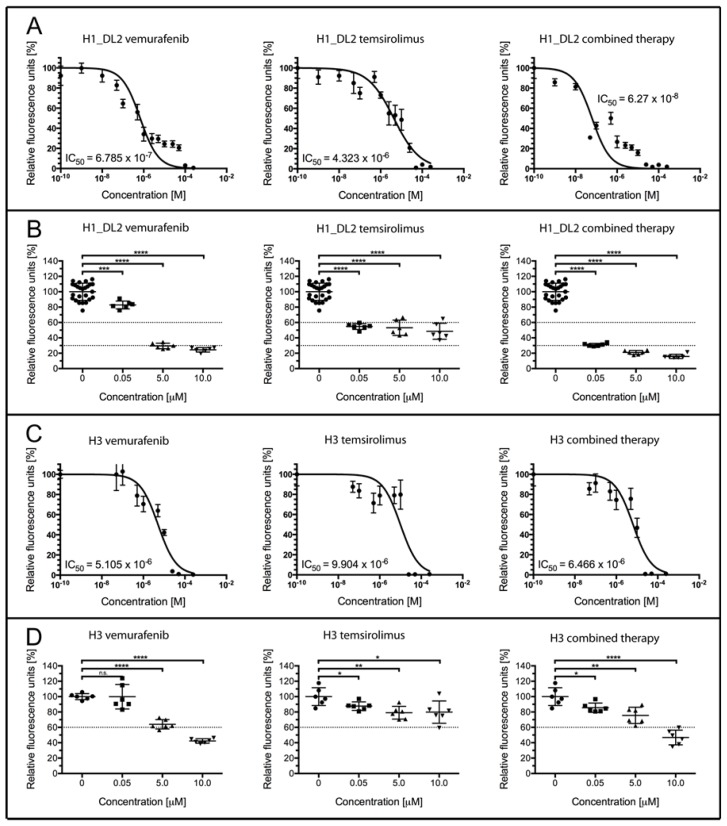
Cell proliferation and survival of H1_DL2 and H3 melanoma brain metastasis cells grown as monolayer cultures, after treatment with vemurafenib and temsirolimus. (**A**,**B**) Treatment of H1_DL2 melanoma cells, harboring the BRAF^V600E^ mutation. (**A**) H1_DL2 cells treated with increasing drug concentrations for 72 h (*n* = 6, mean ± SD); (**B**) A more detailed analysis of the cell survival shown in (A), after drug treatment with 0, 0.05, 5 or 10 μM of vemurafenib, temsirolimus or a combination therapy. Scatter dot plot (*n* = 6, mean ± SD). *** *p* < 0.001; **** *p* < 0.0001; (**C**,**D**) Treatment of H3 melanoma cells, harboring wild-type (WT) BRAF. (**C**) H3 cells treated with increasing drug concentrations for 72 h (*n* = 6, mean ± SD); and (**D**) A more detailed analysis of the cell survival shown in (**C**), after drug treatment with 0, 0.05, 5 or 10 μM vemurafenib, temsirolimus or combined therapy. Scatter dot plot (*n* = 6, mean ± SD). n.s.: not significant; * *p* < 0.05; ** *p* < 0.01; **** *p* < 0.0001.

The H1_DL2 cell line was effectively treated with vemurafenib, with an IC_50_ of 0.679 μM (Figure 1A, left). Treatment with temsirolimus alone was less effective, with an IC_50_ of 4.323 μM (Figure 1A, middle), while combined therapy was shown to be the most effective treatment (IC_50_ = 0.063 μM; Figure 1A, right).

A more detailed comparison of the drug effects on the H1_DL2 cells is shown in Figure 1B and Table 1. At a drug concentration of 0.05 μM, 82.8% of the H1_DL2 cells survived treatment when using vemurafenib, while 54.7% of the cells survived treatment with temsirolimus alone. However, only 31.0% of the cells survived a combined treatment, indicating a synergistic effect of combined therapy (co-efficient of drug interaction (CDI), 0.68; see Table 1). Single drug treatment with vemurafenib was effective at concentrations of 5 or 10 μM (29.5% and 24.4% survival, respectively), while treatment with temsirolimus showed a cell survival of 53.1% (5 μM) and 48.6% (10 μM). Combined treatment was the most effective, with cell survival of 21.0% at 5 μM (synergistic effect) and 15.9% at 10 μM. Pictures of cell survival after treatment with vemurafenib and temsirolimus are also seen in Figure S2. For a comparison, we assessed the treatment effects on the H3 melanoma brain metastasis cell line, which expresses WT BRAF and has a homozygous deletion of PTEN (data not shown). In general, the H3 cells were not as sensitive to therapy as the H1_DL2 cells, where vemurafenib treatment resulted in an IC_50_ of 5.105 μM (Figure 1C, left). Treatment with temsirolimus alone was even less effective, with an IC_50_ of 9.906 μM (Figure 1C, middle). An increased effect using a combined therapy was not seen (IC_50_ = 6.446 μM; Figure 1C, right).

A more detailed comparison of the drug effects on the H3 cells is shown in Figure 1D and Table 2. All H3 cells survived treatment with 0.05 of μM vemurafenib, while 87.6% of the cells survived treatment with temsirolimus alone.

The combined treatment did not increase cell death, as 85.6% of the cells survived treatment. At 5 μM, 64.0% of the cells survived after vemurafenib treatment, while 79.0% of the cells survived when temsirolimus was used alone. Combined therapy at this dose was not more effective (75.6% survival). Similar results were observed at 10 μM (42.3% survival with vemurafenib, 79.8% survival using temsirolimus and 46.8% survival after combined treatment).

### 2.3. Treatment with Vemurafenib Induces Anti-Migratory Effects in the H1_DL2 Cell Line Grown as Monolayers

**Figure 2.**
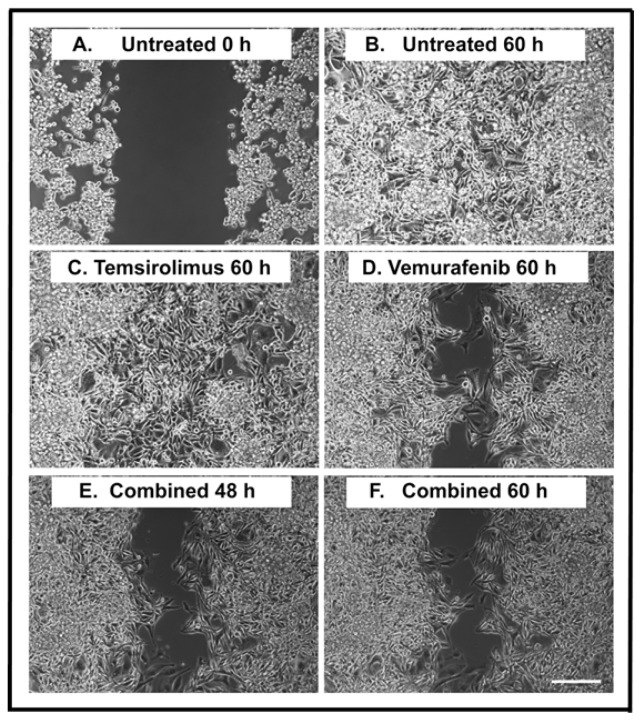
Wound healing assay of H1_DL2 melanoma brain metastasis cells after treatment with vemurafenib and temsirolimus. (**A**) Untreated H1_DL2 cells at 0 h; (**B**) Untreated cells 60 h after initiating a scratch wound; (**C**) Representative picture 60 h after initiating a scratch wound and starting the treatment with 5 μM of temsirolimus; (**D**) Representative picture 60 h after initiating a scratch wound and starting the treatment with 5 μM of vemurafenib; (**E**) Representative picture 48 h after initiating a scratch wound and starting combined treatment (5 μM temsirolimus and 5 μM vemurafenib); and (**F**) Representative picture 60 h after initiating a scratch wound and starting combined treatment (5 μM temsirolimus and 5 μM vemurafenib). Scale bar = 200 μm.

A monolayer wound healing assay was applied to study the effects on cell migration during treatment. By using this assay, we could clearly observe the cell migration of individual tumor cells during a time course of 60 h (Figure 2, and Supplementary Movies 1–4). At 0 h, the width of the open area was around 500 μm (Figure 2A). The wound was closed after 60 h in untreated cells (Figure 2B) and cells treated with 5 μM temsirolimus (Figure 2C). When the H1_DL2 cells were treated with 5 μM of vemurafenib, around 40% of the wound was still open after 60 h (Figure 2D). The combined treatment (5 μM temsirolimus + 5 μM vemurafenib) did not increase the inhibition of cell migration compared to vemurafenib treatment alone (Figure 2E,F).

### 2.4. Treatment of H1_DL2 Spheroids with Vemurafenib and Temsirolimus Inhibits Spheroid Growth and Increases Cell Death

**Figure 3.**
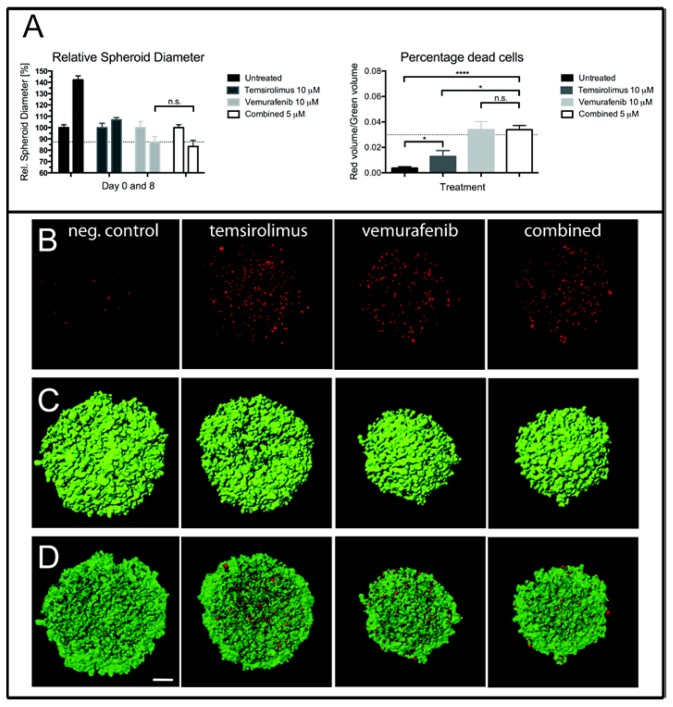
Multicellular spheroid growth of H1_DL2 cells after treatment with vemurafenib and temsirolimus. (**A**, left) Spheroid diameters measured zero and eight days after treatment with 10 μM of temsirolimus, 10 μM of vemurafenib or the combined treatment (5 μM temsirolimus + 5 μM vemurafenib) (*n* = 6, mean ± SD). n.s.: not significant; * *p* < 0.05, **** *p* < 0.0001. (**A**, right) The percentage of dead cells within the spheroids after treatment for three days with 10 μM of temsirolimus, 10 μM of vemurafenib or the combined treatment (5 μM temsirolimus + 5 μM vemurafenib); (**B**) Maximum intensity projection images of the red channel (dead cells) of confocal image stacks of spheroids treated with 10 μM of temsirolimus, 10 μM of vemurafenib or the combined treatment (5 μM temsirolimus + 5 μM vemurafenib); (**C**) The surface volume of the green channel (live cells) of the same spheroids as in (**B**); and (**D**) An overlay of the red and green volume surfaces of the same spheroids as in (**B**). Scale bar = 100 μm. neg. control = negative control.

Spheroid growth was studied by measuring their diameters after Day 0 and 8 (Figure 3A, left, and Figure 3C). There was a 42.1% increase in mean diameter of untreated spheroids from Day 0 to Day 8. Treatment with 5 μM of temsirolimus inhibited spheroid growth compared to the untreated spheroids, as there was an increase in mean diameter of only 6.9% from Day 0 to Day 8. Single drug treatment with vemurafenib was more effective, leading to a 13.1% decrease in diameter from Day 0 to Day 8. Combined treatment reduced the mean diameter even more, as there was a decrease of 16.7% in mean diameter from Day 0 to Day 8, however not statistically significant when compared to vemurafenib treatment alone, *p* = 0.191.

After three days of treatment, the number of dead cells within the spheroids was studied by staining with ethidium homodimer, followed by confocal imaging (Figure 3A right, 3B,D). Vemurafenib (5 μM) increased cell death more effectively than temsirolimus (5 μM) when administered as a single drug, whereas the combined treatment (5 μM of each drug) did not increase the number of dead cells, compared to treatment with vemurafenib alone.

### 2.5. Western Blots Suggest Drug-Related Inhibition of the MAPK (Mitogen-Activated Protein Kinase) and PI3K (Phosphoinositide 3-Kinase) Pathways

**Figure 4.**
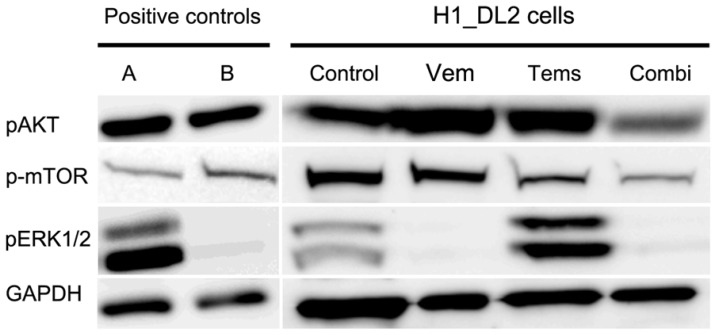
Western blot of H1_DL2 cells after drug treatment with vemurafenib and temsirolimus. Western blot of phosphorylated ERK1 and phosphorylated ERK2 (pERK1/2), phosphorylated mTOR (p-mTOR) and phosphorylated AKT (pAKT), in H1_DL2 cells treated with 15 μM of vemurafenib (Vem), 25 μM of temsirolimus (Tems), or the combined treatment (Combi; 10 μM vemurafenib + 10 μM temsirolimus). The K16 cell line (**A**) served as positive control for pMAPK; and the 293T cell line (**B**) was a positive control for pAKT and p-mTOR. GAPDH protein levels were assessed as a loading control.

Untreated H1_DL2 cells expressed pAKT, p-mTOR and pERK1/2, as shown by western blots (Figure 4). The expression of phosphorylated ERK1/2 disappeared after treatment with 15 μM vemurafenib, whereas no change was found in cells treated with temsirolimus. Treatment with 25 μM temsirolimus reduced the expression of p-mTOR, and the combined treatment (10 μM vemurafenib + 10 μM temsirolimus) inhibited the expression of phosphorylated ERK1/2 and reduced the expression of p-mTOR and pAKT.

### 2.6. Principal Component Analysis of Gene Expression Arrays Shows Alterations in Gene Clustering after Treatment

**Figure 5.**
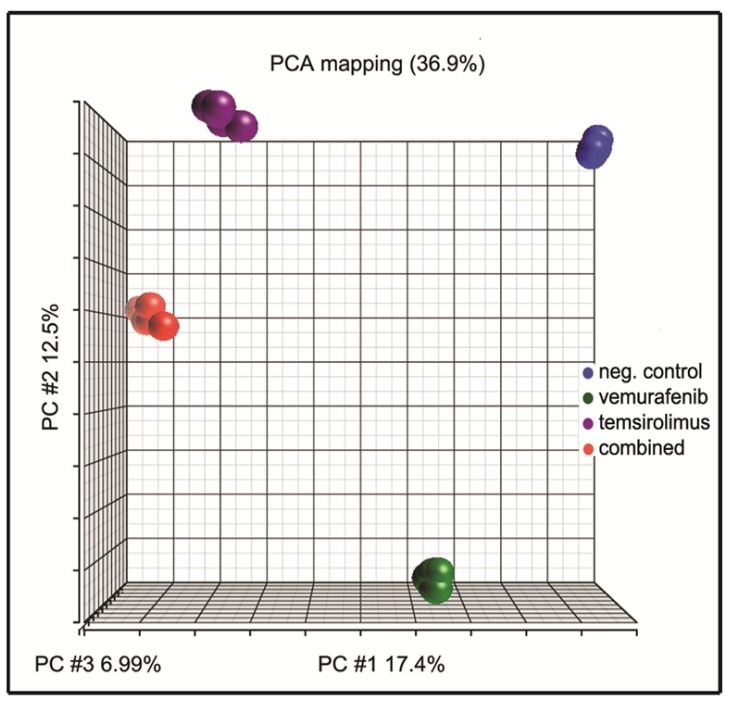
Principal component analysis (PCA) of H1_DL2 cells after drug treatment with vemurafenib and temsirolimus. PCA analysis of all 20 samples resulted in four distinct clusters for each of the respective treatments: Untreated samples (blue), vemurafenib-treated cells (15 μM; green), temsirolimus-treated cells (25 μM; purple) and the combination treatment (5 μM each; red) (*n* = 5 for all treatments).

Principal component analysis (PCA) is an unsupervised global analysis approach and is useful for reducing the dimensionality of gene expression data. PCA allows for projecting multidimensional gene data onto just two or three new dimensions that explain most of its variance. As a result, it is possible to visually interpret major trends in the data, for example, in terms of the similarity between various data points [32]. The PCA of our gene expression data showed that the different samples separated well into tightly pooled clusters for each of the respective treatments, without any outliers (Figure 5).

### 2.7. Pathways Affecting Cell Cycle Checkpoint Regulation Are Affected by Combined Treatment

**Figure 6.**
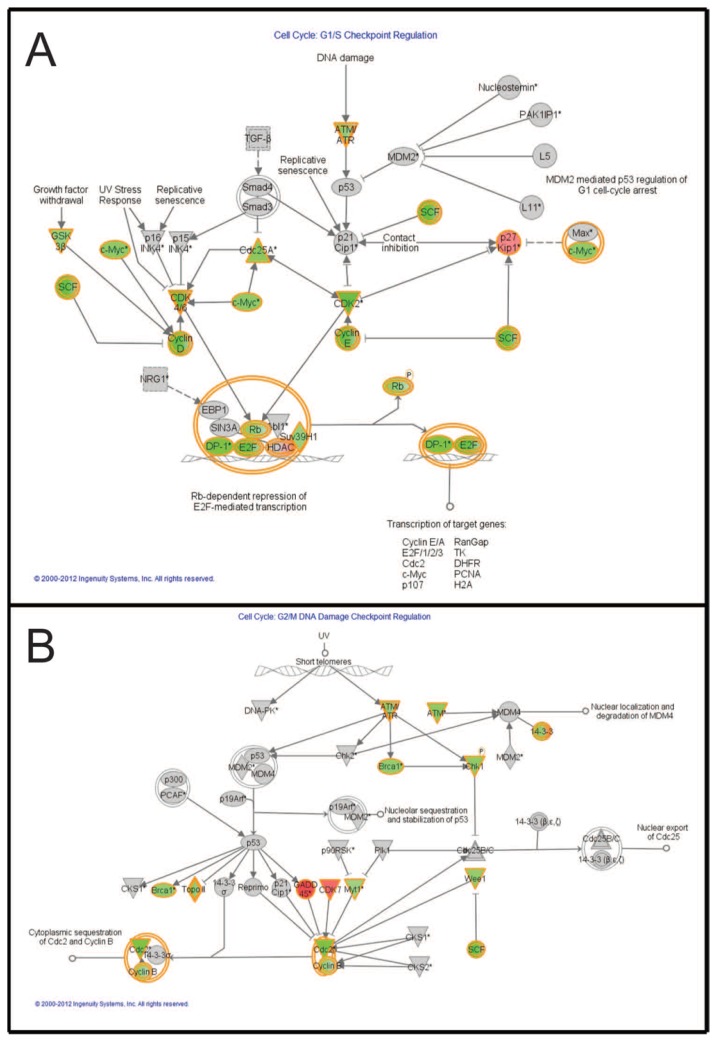
Graphical representation of two of the top score networks identified by Ingenuity Pathway Analysis (IPA). Canonical pathways from Ingenuity for combination treatment (5 μM vemurafenib and 5 μM temsirolimus) compared to untreated cells. Molecular relationships between genes downregulated (green) or upregulated (red) after treatment are shown. (**A**) The canonical pathway “Cell cycle: G1/S checkpoint regulation”; and (**B**) The canonical pathway “Cell cycle: G2/M DNA damage checkpoint regulation”.

Functional analysis of negatively or positively regulated genes before and after combination treatment was performed using Ingenuity Pathway Analysis (IPA; [33]). Two of the top pathways were “Cell cycle: G1/S checkpoint regulation” (Figure 6A) and “Cell cycle: G2/M DNA damage checkpoint regulation” (Figure 6B), where a number of genes in the pathways were downregulated after combined treatment (5 μM vemurafenib and 5 μM temsirolimus).

### 2.8. Several Genes Related to Cell Survival and Invasion Are Affected by Treatment

Based on the *z*-score, showing whether potential downstream functions were “activated” (a *z*-score larger than two) or “inhibited” (a *z*-score less than −2), a list of 17 downstream functions could be selected (Table 3; *p* ≤ 0.005).

**Figure 7.**
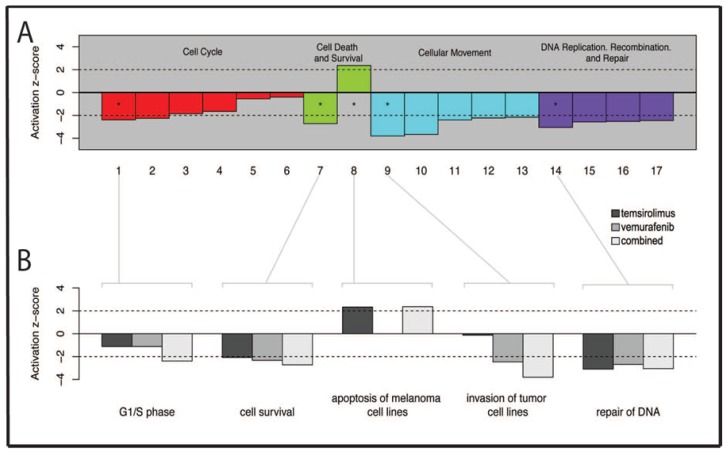
Ingenuity function analysis. (**A**) Bar plot of the activation *z*-scores of selected downstream functions in combination treated samples (5 μM vemurafenib and 5 μM temsirolimus) compared to untreated cells. Functions with *z*-scores greater than two are predicted as being “activated” in this comparison, while those with scores less than −2 are “inhibited”. All columns were statistically significant; *p* < 0.005. Column numbers correspond to the list of downstream functions shown in Table 3; and (**B**) A detailed presentation of bar plots of activation *z*-scores, showing the most activated downstream functions for each of the three treatments; 25 μM temsirolimus *vs*. untreated cells, 15 μM vemurafenib *vs.* untreated cells or 5 μM vemurafenib + 5 μM temsirolimus *vs.* untreated cells. All columns were statistically significant; *p* < 0.005.

These functions were categorized into the following groups: cell cycle; cell death and survival; cellular movement and DNA replication; and DNA recombination and DNA repair (Figure 7A). Furthermore, to evaluate the effects of the different treatments (vemurafenib *vs.* control, temsirolimus *vs.* control and combination *vs.* control), five of the most activated functions from each of the major groups were studied in more detail (Figure 7B). Overall, the microarray analysis indicated that the inhibitory effects of treatment was related to major cellular functions within the H1_DL2 cells, such as the downregulation of genes associated with the cell cycle, cellular movement and DNA replication, DNA recombination and DNA repair and upregulation of genes associated with cell death.

### 2.9. Discussion

Melanoma patients with advanced disease have a high chance of developing brain metastases, which is associated with a poor prognosis. Apart from introducing Gamma Knife radiosurgery, conventional treatment of these metastases has not changed during the last two decades [3], necessitating the development of new therapeutic strategies. Several small molecule inhibitors targeting signaling pathways have been developed recently. However, with a few exceptions [24,27,28], they have not been studied clinically on patients with brain metastases from melanoma.

The two specific protein kinase inhibitors, vemurafenib [34–36] and temsirolimus [2,37,38], have previously been shown to target the MAPK and PI3K pathways, respectively. Lazar and colleagues showed that more than 10% of human melanomas display a loss of PTEN expression, in addition to BRAF^V600E^ mutation [39]. PTEN is one of the critical negative regulators of AKT activity [40]. Thus, the loss of PTEN expression leads to increased PI3K/AKT signaling, particularly when BRAF is inhibited, which, in turn, may contribute to an intrinsic resistance of BRAF^V600E^ mutated melanoma cells to vemurafenib treatment [17]. Thus, it is clear that single drug therapy targeting individual pathways may be insufficient to obtain therapeutic efficacy. The fact that melanomas develop resistance to vemurafenib emphasizes the importance of targeting multiple pathways simultaneously [9].

We set out to study whether the growth and proliferation of human melanoma brain metastasis cells could be inhibited more efficiently *in vitro* by combining therapies targeting two major pathways in melanoma progression. To our knowledge, this study is the first to demonstrate inhibitory effects on melanoma brain metastatic cell lines by combining vemurafenib and temsirolimus, and we show the regulation of genes after treatment that may be involved in reducing the invasive potential of melanoma cells.

The BBB has an important role in the treatment of brain metastasis. The delivery of chemotherapy is usually ineffective, due to an often intact vascular barrier in the brain with low passive transcellular permeability. Furthermore, the endothelial cells of the brain vasculature express active efflux drug transporters, which limit the uptake of most anticancer drugs. We have previously shown in an animal model of brain metastasis that the BBB is relatively leaky for small contrast agents (molecular weight (MW) below 300 Da) at early stages and permeable to magnetic resonance imaging (MRI) contrast agents (MW 0.566 and 2.066 kDa) at later stages of brain metastatic development [4]. These results suggest that small MW drugs, such as vemurafenib and temsirolimus, could potentially cross the BBB early in the development of brain metastases.

By targeting BRAF and mTOR simultaneously, we were able to inhibit the cell growth and proliferation (Figure 1) of the H1_DL2 cells in monolayers. Combined treatment resulted in a synergistic increase in cell death compared to single drug treatment (at concentrations of 0.05 and 5 μM), as shown by the resazurin assay (Table 1). However, temsirolimus alone was not able to decrease the migratory capacity of cells grown as monolayer cultures, compared to untreated cells. These results were supported by our gene analysis data, indicating minimal effects on genes related to invasive capacity after single dose treatment with temsirolimus (Figure 7B; Group 9). The gene analysis, however, indicated that combined therapy was more effective at inhibiting cell invasion, compared to single-dose treatment with vemurafenib.

Assays using tumor cells grown as multicellular spheroids are regarded as more relevant with respect to solid tumor growth seen *in vivo* [41]. In our study, vemurafenib was more effective than temsirolimus in inhibiting spheroid growth *in vitro*, and the combined therapy suggested an additional growth inhibition. The number of dead cells within the spheroids did not increase after combined treatment compared to vemurafenib treatment alone, which likely can be explained by the previously observed poor penetrance of ethidium homodimer into compact spheroid structures. Our results on monolayer cells and spheroids, taken together, indicate that combined therapy may inhibit single tumor cell growth, as well as when treating solid tumors, provided that the drugs would effectively cross the BBB. To study this further, upcoming animal studies will investigate the effects of single and combined treatment on brain metastasis, both at the single-cell level in the animal brain using iron oxide prelabeling of the tumor cells [31] and on solid tumor development.

The western blots showed that the expression of pMAPK was lost after combined treatment, and the expression of p-mTOR pAKT was reduced, suggesting that the MAPK and PI3K signaling pathways were inhibited.

Gene expression profiling is important for the identification of genes that may be involved in tumor progression and metastasis and also for studying how tumor cells respond to therapy. In addition, gene expression profiling has been important for a molecular sub-classification of tumors [42,43]. PCA is an exploratory visualization of gene expression data and discriminates between sample groups [32]. In our treatment study, PCA separated the sample groups for each respective treatment (Figure 5) into tightly pooled clusters well.

A functional analysis of negatively or positively regulated genes before and after combination treatment was performed using IPA. Interestingly, pathways representing cell cycle regulation were downregulated after combined treatment of our melanoma brain metastasis cells (Figure 6).

Further analysis of these pathways using the activation *z*-score (Figure 7A and Table 3) showed seventeen downstream functions that were either activated (*z*-score > 2) or inhibited (*z*-score < −2) by combined treatment. These functions could be grouped into four different categories: (1) cell cycle; (2) cell death and cell survival; (3) cellular movement; and (4) DNA replication, DNA recombination and DNA repair. We then performed a more detailed analysis of the downstream functions with the highest relative *z*-score, to study the differences in gene expression after single drug treatment and combined treatment. Gene sets related to G1/S phase, cell survival and cell invasion showed larger effects on gene expression after combined treatment, compared to the single drug treatments (Figure 7B). Interestingly, vemurafenib treatment did not show any effects on downstream functions related to the apoptosis of melanoma cell lines, while DNA repair mechanisms resulted in low action scores (<−2) irrespective of treatment. Based on our functional analysis, further work is warranted to verify key genes that may represent therapeutic targets. This was, however, beyond the scope of this study.

## 3. Materials and Methods

### 3.1. Cell Line and Cell Culture

The H1 and H3 cell lines were established in our laboratory from patient biopsies of human melanoma brain metastasis, as previously described [31,44]. Written consents were obtained from the patients before tumor material was collected. The Regional Ethical Committee (REC Number 013.09) and the Norwegian Directorate of Health (NSD Number 9634) approved the tissue collection and biobank storage of tumor biopsies and derived cell lines. The cells were authenticated in February, 2013, using the AmpFℓSTR^®^ Profiler Plus^®^ *ID* PCR Amplification Kit (Applied Biosystems Inc., Foster City, CA, USA), and short tandem repeat (STR) profiles were matched to the parent tumor and cross-checked with cell line profiles at [45].

The H1 cells were transduced with two lentiviral vectors, encoding Dendra (a green fluorescent protein (GFP) variant) and luciferase to obtain the H1_DL2 cell line. Flow cytometric isolation of cells by GFP expression was performed (BD FACSAria, Becton Dickinson, Franklin Lakes, NJ, USA).

The cell lines were grown in DMEM supplemented with 10% heat-inactivated newborn calf serum, four times the prescribed concentration of non-essential amino acids, 2% l-glutamine, penicillin (100 IU/mL) and streptomycin (100 μL/mL) (Cambrex, East Rutherford, NJ, USA). The cells were incubated in a standard tissue culture incubator at 37 °C, 100% humidity and 5% CO_2_.

### 3.2. BRAF Gene Mutation Status

The H1_DL2 and the H3 cell lines were checked for BRAF^V600E^ mutation status by Sanger dideoxy sequencing of PCR amplicons from genomic DNA using primers, as described previously [1]. PCR amplicons were purified by ExoSapI (Affymetrix, Santa Clara, CA, USA) before sequencing, using the BigDye^®^ Terminator v 3.1 Cycle Sequencing Kit (Invitrogen, Carlsbad, CA, USA).

### 3.3. PTEN Deletion Status

The PTEN deletion status in the H1_DL2 and H3 cell lines was studied by DNA copy number analysis. Genomic DNA was extracted from cell lines using the DNAeasy Blood and Tissue Kit (Qiagen GmbH, Hilden, Germany) following the manufacturer’s instructions. DNA was eluted in water, fragmented to an average size of 200–500 bp using DNAse1 (rDNAse1, Ambion, Life Technology Ltd., Grand Island, NY, USA) and labeled using the BioPrime aCGH Genomic Labeling Kit (Invitrogen, Carlsbad, CA, USA) and Cy3 and Cy5 dyes purchased from GE Healthcare (Chalfont St. Giles, UK), following standard protocols for Agilent aCGH. Commercially available female DNA pooled from multiple anonymous donors (Promega, Madison, WI, USA) was used as a reference. Labeled DNA was competitively hybridized to SurePrint G3 Human 2 × 400 K CGH microarrays (Agilent Technologies, Santa Clara, CA, USA) following standard Agilent protocols. The slides were scanned at 3-μm resolution using the Agilent High-Resolution Microarray scanner and the image data were extracted using feature extraction (Agilent Technologies). FE extraction files were imported into Genomic Workbench (Agilent Technologies) for visualization and analysis. Aberrations were called using the ADM2 algorithm with a threshold setting of 25, centralization on, with a threshold of 25 and an aberration filter min Probes = 5 and min AvgAbsLogRatio = 0.45.

### 3.4. In Vitro Drug Treatment

Vemurafenib was purchased from ChemieTek (Indianapolis, IN, USA) and temsirolimus from Sigma-Aldrich (St. Louis, MO, USA). Both drugs were dissolved in dimethylsulfoxide (DMSO) and stock concentrations of 50 mM were stored at −20 °C in aliquots until use. Final concentrations were made in growth medium (0, 0.001, 0.01, 0.05, 0.1, 0.5, 1, 2.5, 5, 10, 25, 50, 100 or 250 μM), before adding to the cell monolayers.

### 3.5. Resazurin Cell Proliferation Assay

Cell proliferation before and after treatment (vemurafenib, temsirolimus or combined treatment) was studied using a resazurin assay, as described previously [46]. Briefly described, 4000 H1_DL2 or H3 cells in 100 μL cell culture medium were seeded into each well of a 96-well plate and cultured for 24 h. The cell monolayers were then subsequently treated with drugs (at concentrations described in Section 3.4) for 72 h. After treatment, the culture medium containing drugs was removed, and fresh culture medium with 10 μg/mL resazurin (Sigma-Aldrich) diluted in phosphate buffered saline (PBS) was added. Following incubation for 4 h, the absorbance was measured at dual mode 560/590 nm with a scanning multiwell spectrophotometer (Victor 3 1420 multi-label counter, Perkin Elmer, Waltham, MA, USA), using WorkOut v2.0 software (Dazdaq Solutions Ltd., East Sussex, UK). The results were prepared in GraphPad Prism v6 software (GraphPad Software, Inc., La Jolla, CA, USA).

IC_50_ was defined as the drug concentration at which 50% of the cell growth was inhibited. The calculations of IC_50_ were performed in GraphPad Prism v6. The co-efficient of drug interaction (CDI) was calculated using the formula: CDI = AB/(A × B). According to the absorbance of each group, AB is the ratio of the combination group to the control in OD 560/590, and A or B is the ratio of the single drug group to the control group in OD 560/590. CDI < 1 indicates synergism; CDI < 0.7 indicates significantly synergistic effect; CDI = 1 indicates additivity; and CDI > 1 indicates antagonism, as described previously [47].

### 3.6. Cell Migration Assay

To study the effects of treatment on cell migration, a monolayer wound healing assay was performed. A silicone culture insert (Ibidi GmbH, Martinsried, Germany) was inserted into each well of a 4-well μ-Slide (Ibidi GmbH), and 70 μL of H1_DL2 cells (concentration: 9 × 10^5^ cells/mL in cell culture medium) were added into each half of the culture inserts. The cells were allowed to grow to confluence for 24 h, before the culture insert was removed, resulting in a wound (a cell free gap) with a width of 500 μm within the culture. Drugs were then added into the wells (5 μM vemurafenib, 5 μM temsirolimus or 5 μM vemurafenib + 5 μM temsirolimus), and cell migration into the wound was studied by time lapse microscopy for 60 h, using a Nikon TE2000 inverted microscope (Nikon Instruments Inc., Melville, NY, USA) equipped with an incubator holding 37 °C, 100% humidity and 5% CO_2_. Pictures were then processed and analyzed with Image-Pro Plus (MediaCybernetics, Warrendale, PA, USA). The experiments were performed in triplicate.

### 3.7. Spheroid Growth and Cytotoxicity

Multicellular spheroids from the H1_DL2 cells were prepared using a protocol modified from Ivascu and Kubbies [48]. Four thousands cells in 100 μL of cell culture medium were distributed per well in a Costar 96-well round bottom ultra-low attachment plate (Corning Inc., Corning, NY, USA). The plate was then centrifuged at 1000× *g* for 15 min and returned to the cell incubator for 5 days. At that time, the cells had formed spheroids with mean diameters of around 700 μm.

The spheroids were exposed to drugs for 8 days, either as single drugs (10 μM) or as a combined treatment (5 μM vemurafenib + 5 μM temsirolimus). Two orthogonal diameters were measured for each spheroid after 0 and 8 days with a Nikon TE2000 inverted microscope using the 4× objective, and the mean spheroid diameters (±SD) were calculated in GraphPad Prism v6.

The treatment effects on cells within the spheroids were evaluated at Day 3 using a Live/Dead Viability/Cytotoxicity Kit (Molecular Probes, Eugene, OR, USA), by staining the spheroids with 2 μM ethidium homodimer for 30 min at room temperature. The number of live cells (expressing GFP) and dead cells (stained red by ethidium homodimer) was visualized by confocal microscopy. For each treatment, three spheroids were studied, and both the acquisition of images and the analysis were performed with the exact same parameters for all spheroids studied. z-stacks of 50 optical sections through a total depth of 100 μm were acquired with an inverted Leica TCS SP5 confocal microscope (Leica Microsystems, Wetzlar, Germany) using a HCX PL APO CS 10 × 0.4 NA objective and further processed in Imaris version 7.6.3 (Bitplane AG, Zurich, Switzerland). Surface rendering was performed on both the red and green channel. Threshold levels for the red channel were set to visualize single cells, whereas the threshold level for the green channel was set to measure the total volume of the spheroid. Only red cells that were surrounded by the green volume were accounted for in the statistical analysis. The result is presented as the number of dead cells (red) per spheroid unit volume (green) and was prepared in GraphPad Prism v6.

### 3.8. Western Blot Analysis

H1_DL2 cell monolayers were grown in T75 culture flasks (Nunc, Roskilde, Denmark) until the monolayers were 70%–80% confluent. The cells were then treated with drugs (15 μM vemurafenib, 25 μM temsirolimus or 10 μM vemurafenib + 10 μM temsirolimus) and incubated for 72 h. The cells were washed twice with ice-cold phosphate buffered saline and lysed in 450 μL Kinexus lysis buffer (Table S1). The samples were harvested with a sterilized cell scraper, homogenized using a Kontes pellet pestle motor (Fisher Scientific), sonicated for 4 × 10 s (Bandelin Sonorex RK255S) and centrifuged at 10,000× *g* for 10 min at 4 °C. Aliquots of supernatant were stored at −80 °C until further analysis. Protein concentration of the cellular extracts was determined using the Bio-Rad Bradford assay (Bio-Rad Laboratories, Inc., Berkeley, CA, USA), as per the kit instructions, using bovine serum albumin as the standard. 20 mg of protein extract were electrophoresed on 4%–12% NuPage Bis-Tris-Glycine gels (Life Technologies, Carlsbad, CA, USA) for 50 min at 200 V. Proteins were blotted onto nitrocellulose membranes (Whatman, Germany) at 30 V for 90 min using a tank blot system. The membranes were blocked with 0.05 g Defco dry milk powder per mL of blocking solution (TBST buffer; 0.02 M Tris-HCl (pH 7.5), 0.15 M NaCl and 0.1% Tween 20) for 1 h at 4 °C. Immunostaining was performed using antibodies against p-AKT (Ser473) (dilution: 1:500), p-mTOR (Ser2448) (dilution: 1:500), p-p44/42 MAPK (ERK1/2) (Thr202/Tyr204) (dilution: 1:2,000) (Cell Signaling, Danvers, MA, USA) and GAPDH (dilution: 1:2,500) (Abcam, Cambridge, UK) diluted in blocking solution and incubated overnight at 4 °C. A glioblastoma xenograft, as described previously [49], was used as the positive control for pMAPK (noted as A in Figure 4), and the 293T cell line (ATCC # CRL-11268) served as a positive control for pAKT and p-mTOR (noted as B in Figure 4). The membranes were washed 4 times for 10 min each before being exposed to secondary antibody, anti-rabbit HRP (Beckman Coulter, Brea, CA, USA), diluted 1:20,000 in blocking buffer and incubated for 90 min at room temperature. Bound antibody was visualized by using a chemiluminescence detection system, SuperSignal West Femto, and imaged by LAS3000.

### 3.9. RNA Extraction after Drug Treatment of Monolayer Cultures

Total RNA was extracted from cell samples by the RNeasy Plus Kit (Qiagen). The H1_DL2 cells were grown in a 6-well plate (Nunc) with 5 parallels for each sample. All samples were treated and harvested at the same time. When confluent, cell monolayers were exposed to either 15 μM vemurafenib, 25 μM temsirolimus or a combination of 5 and 5 μM temsirolimus for 24 h. These cells were then washed once with PBS, followed by the addition of 350 μL RLT lysis buffer into each well. The cells were then removed using a cell scraper. The remaining procedure was followed according to the manufacturer’s instructions, including DNase 1 solution treatment (Qiagen). RNA samples were stored at −80 °C until further analysis.

### 3.10. Illumina HT-12 Array; RNA Preparation, Labeling and Microarray Hybridization

All microarray experiments were performed using the Illumina iScan Reader (Illumina, Inc. San Diego, CA, USA), which is based upon fluorescence detection of biotin-labeled cRNA. 500 ng of total RNA from each sample were reversely transcribed, amplified and biotin-16-UTP-labeled, using the Illumina TotalPrep RNA Amplification Kit (Life Technologies, Carlsbad, CA, USA). The amount and quality of the Biotin-labeled cRNA was controlled using a NanoDrop spectrophotometer (Nanodrop Products, Wilmington, DE, USA) and an Agilent 2100 Bioanalyzer (Agilent Technologies, Santa Clara, CA, USA). 750 ng of biotin-labeled cRNA were hybridized to the HumanHT-12 V4 Expression BeadChip (Illumina, San Diego, CA, USA), according to the manufacturer’s instructions. The HumanHT-12 V4 BeadChip targets 47,231 probes derived primarily from genes in the NCBI RefSeq database (Rockville, MD, USA) (Release 38).

### 3.11. Microarray Analysis

Principal component analysis and analysis of variance were performed using Partek Genomics Suite (Partek Inc., St. Louis, MO, USA). The gene expression data was projected onto 3 principal components (PC 1, 2 and 3) and the projections were used for an exploratory visualization of the data. Results were significant if the absolute fold change was more than 1.6 or less than −1.6, with a *q*-value < 0.001. Ingenuity’s Pathway Analysis (IPA) (Ingenuity^®^, [33]) was used for pathway and functional analysis. Plots were made using the R language v.3.0.1 (The R Foundation for Statistical Computing, Vienna, Austria).

### 3.12. Statistics

The statistical significance of differences was evaluated by an unpaired two-tailed *t*-test, using GraphPad Prism v6. Values presented in the figures represent means ± standard deviation. A two-tailed *p* ≤ 0.05 was considered to be statistically significant.

## 4. Conclusions

In summary, we show that treatment of BRAF^V600E^-mutated melanoma brain metastases cell lines may be effectively treated *in vitro* with vemurafenib combined with temsirolimus. Our results described here should be further verified in animal experiments, which could lead to new knowledge to be used in future clinical trials.

## Figures and Tables

**Table 1.** Tumor cell survival after treatment of H1_DL2 melanoma brain metastasis cells. CDI, coefficient of drug interaction.

Concentration (M)	Temsirolimus (% Survival)	Vemurafenib (% Survival)	Combined (% Survival)	CDI
0	100 ± 2.2	100 ± 2.2	100 ± 2.2	-
0.05	54.7 ± 1.5	82.8 ± 2.1	31.0 ± 0.8	0.68
5	53.1 ± 4.0	29.5 ± 1.5	21.0 ± 1.1	0.66
10	48.6 ± 4.2	24.4 ± 1.2	15.9 ± 1.1	1.34

**Table 2.** Tumor cell survival after treatment of H3 melanoma brain metastasis cells. CDI, coefficient of drug interaction.

Concentration (M)	Temsirolimus (% Survival)	Vemurafenib (% Survival)	Combined (% Survival)	CDI
0	100 ± 4.7	100 ± 4.7	100 ± 4.7	-
0.05	87.6 ± 2.3	99.9 ± 6.5	85.6 ± 2.5	0.98
5	79.0 ± 3.4	64.0 ± 2.5	75.6 ± 4.3	1.50
10	79.8 ± 5.9	42.3 ± 1.2	46.8 ± 3.9	1.39

**Table 3.** Specification of the downstream functions seen in Figure 7.

Bar Number	Downstream Functions
1	G1/S phase
2	S phase
3	M phase
4	G1 phase
5	G2/M phase
6	G2 phase
7	Cell survival
8	Apoptosis of melanoma cell lines
9	Invasion of tumor cell lines
10	Cell movement of tumor cell lines
11	Chemotaxis of PBMCs
12	Cell movement of carcinoma cell lines
13	Cytokinesis of tumor cell lines
14	Repair of DNA
15	Checkpoint control
16	Alignment of chromosomes
17	Chromosomal congression of chromosomes
